# Murderers or thieves at risk? Offence-related suicide rates in adolescent and adult prison populations

**DOI:** 10.1371/journal.pone.0214936

**Published:** 2019-04-03

**Authors:** Daniel Radeloff, Franziska Stoeber, Thomas Lempp, Mattias Kettner, Katharina Bennefeld-Kersten

**Affiliations:** 1 Department of Child and Adolescent Psychiatry, Psychotherapy and Psychosomatics, University Hospital Leipzig, Leipzig, Germany; 2 Department of Child and Adolescent Psychiatry, Psychosomatics and Psychotherapy, Goethe University Frankfurt, Frankfurt, Germany; 3 Institute of Forensic Medicine, Goethe University Frankfurt, Frankfurt, Germany; 4 Institute for Suicide Research, Restorf-Hoebeck, Germany; University of Bern, SWITZERLAND

## Abstract

**Purpose:**

Prisoners have a higher risk of suicide compared to non-incarcerated individuals. One aim of suicide prevention for prisoners is to identify risk factors in order to put stronger support mechanisms in place for the more vulnerable detainees. This study investigates the suicide risk (SR) in offence-related sub-populations in a representative German sample and differentiates between SR for adolescent and adult prisoners.

**Methods:**

Conducting a national study with data from public German records on the entire prison population from 2000 to 2016 and suicide numbers in German prisons in the same period, SR was calculated for the total male prison population as well as for both subgroups, adolescent and adult male prisoners.

**Results:**

In the study period, male prisoners spent 959.584 life years (LY) in German criminal detention. Among those, 524 prisoners died of suicide. SR was higher for detainees imprisoned for an offence resulting in extensive physical harm for another person, e.g. homicide (suicide rate = 134,8 suicides per 100.000 LY; OR = 2,47; CI95%: 1,98–3,08), bodily injury (suicide rate = 87,3; OR = 1,60; CI95%: 1,29–1,99), and sexual offences (suicide rate = 84,2; OR = 1,54; CI95%: 1,18–2,01) compared with the SR of the total prison population (suicide rate = 54.6). Age differences between offence-related SR were found for theft, with adolescents (suicide rate = 69,3; OR = 1,25; CI95%: 0,85–1,84) showing higher SR than adults (suicide rate = 38,2; OR = 0,7; CI95%: 0,54–0,92).

**Conclusion:**

The index offence of detainees is associated with SR and age-related differences exist. Suicide prevention in prisons should take both into account to determine populations at risk.

## Introduction

Suicide prevention is a public health imperative [1] and according to WHO guidelines, it is crucial that in the course of public health suicide prevention approaches high-risk groups are identified and psychiatric support is provided [2].

### Suicide and delinquency

Several large epidemiological studies and reviews show an association of delinquent behaviour and suicide [3–7]. Especially, detained individuals are at a high risk for suicide: national total surveys in Great Britain and Germany reported higher suicide risks (SR) for prisoners compared with the general population [8–10]. Three main aspects are discussed to have an influence on elevated SR in detainees. First, individual risk factors that may apply to detainees even before imprisonment. These can be prior suicide attempts, misuse- or dependence of drugs, psychiatric disorders and impulsive character traits [11,12]. Moreover, suicide risk is associated with male gender in the general population and males are clearly over-represented in prison populations [12]. Second, in addition to this imported risk-profile detainees are confronted with stressors that constitute environmental risk factors. These can be associated with prison characteristics like e.g., overcrowding and high turnover [13], perceived lack of control, isolation from family and significant other, fear of the unknown, distrust of an authoritarian environment, perceived lack of control over the future, shame or guilt over the alleged offences [14]. Third, imprisonment may exacerbate a momentary crisis situation, which may act as a situational risk factor (e.g. committal to prison, pronouncement of sentencing).

Individual, environmental, and situational risk factors are likely to differ between offence-related sub-populations. For example, the prevalence of psychiatric disorders differs between offence groups [15]. The association between suicide risk and index offence was analysed in few studies most of which found an increased SR for detainees imprisoned for violent offences like homicide or sexual offences [7,14,16,17]; for an overview on risk factors see [18]. However, the available empirical studies on SR in prisoners in relation to offence type are heterogeneous and so far a differentiation and comparison of adult and adolescent sub-samples has not yet been reported.

### Suicide in adolescent prisoners

While suicidal phenomena in adolescents are described as relatively common [19] and actual rates remain lower than in adults [20] suicide constitutes one of the leading causes of death in young people [19]. Suicide rates among young inmates have been reported for the US-American prison system [14,21] as well as for British [9,10,22], German [23], Italian [24] and Swedish [3] national samples. Findings on adolescent suicides in prison show higher SR and differing risk factors for imprisoned adolescents than for imprisoned adults [10,23]. Several studies found significantly higher suicide rates in adolescents prisoners compared to the age-adapted general population [21,24,25].

### Research questions and hypotheses

In the present study, we aim to investigate the association between SR and offence type for a total national German sample of detainees and extend the scope of the analyses by differentiating between adolescent and adult prison populations. Hereby we will address the following hypotheses: 1) Offence-related sub-populations show significantly different SR with highest SR in homicide and other violent offences; 2) the offence-related SR of adolescents differ from those of adults.

## Methods

### Sample

The total male population of detainees in German criminal detention between 2000 and 2016 was included into the analysis. Moreover, this study includes all cases of completed suicides in males conducted in German criminal detention in the same study period. The investigation is limited to male prison suicides, as men represent approximately 95% of the prison population. Data were obtained for age strata 14–18 years, 18–21 years, 21–25 years and 25 years + and grouped according to index offence. The age group 18–25 years (adolescents) were combined to allow a statistical evaluation and compared with age group 25 years+ (adults). In this study, we apply the terminology adolescents in its broad definition covering both, minors and young adults. Suicides were set in relation to data on the prison population given in life years (LY) for the same time interval.

### Data acquisition and procedure

Numbers of prisoners in German criminal detention and their demographical and criminological characteristics data were provided by the annual census of the Federal Statistical Office Germany. Suicide incidents were retrospectively collected on a national basis by one author (KBK). Every federal state is obliged to report all suicides in detention, based on *reports of unusual events* of all criminal detentions. Criminological information on each suicide was requested from criminal detentions. This data was extracted from individual prison records and given to the authors in an anonymous form.

The majority of detainees are convicted for multiple offences. Therefore, in all datasets used, detainees were grouped according to their index offence, defined as the offence with the highest severity of penalties by law. All major offences that accounted for at least 5% of the prison population were included into the analysis. In accordance with definitions given by German law, offences were classified into one of nine groups: 1) offences against life, e.g., homicide, 2) offences against sexual self-determination, e.g., sexual abuse, rape, 3) offences against physical integrity, e.g., grievous bodily harm, 4) robbery and extortion, 5) theft, 6) fraud, 7) offences related to violations of the narcotics law, 8) traffic related offences and 9) other offences.

### Analytical strategy

Data were analysed with IBM SPSS 24.0 [26], Microsoft Excel, and the R software package 3.4.3 [27]. In order to test hypothesis 1, 2x2 tables were given with suicide numbers and life years (LY) for each offence-related sub-population and suicide numbers and LY of the total prison population (reference population). Hypothesis 1 was tested by calculating odd's ratios (OR) with 95% confidence intervals (CI95%) and Chi-Squares. Moreover, OR were calculated for each combination of offences in direct comparison. To calculate CI95%, approximations using the natural logarithm were applied [28]. In order to test hypothesis 2, suicide numbers and LY were identified for each offence related sub-population and age group. The Tarone test of homogeneity [29] was performed to determine whether the association between age group and suicide (2x2) differed between offence related sub-populations. Poc-hoc, offence related SR of both age groups were set into relation by calculating OR, CI95% and Chi Square values for each offence group. Due to data structure, it was not possible to control for confounding variables (e.g. age, sentence length, nationality, prison conditions, prevalence of psychiatric disorders, social connectedness).

### Ethical considerations

The study was approved by the ethics committee of the medical faculty of the University Hospital Leipzig, Germany and conducted according to the declaration of Helsinki.

## Results

### Suicides and LY in criminal detention

**Fig 1:**
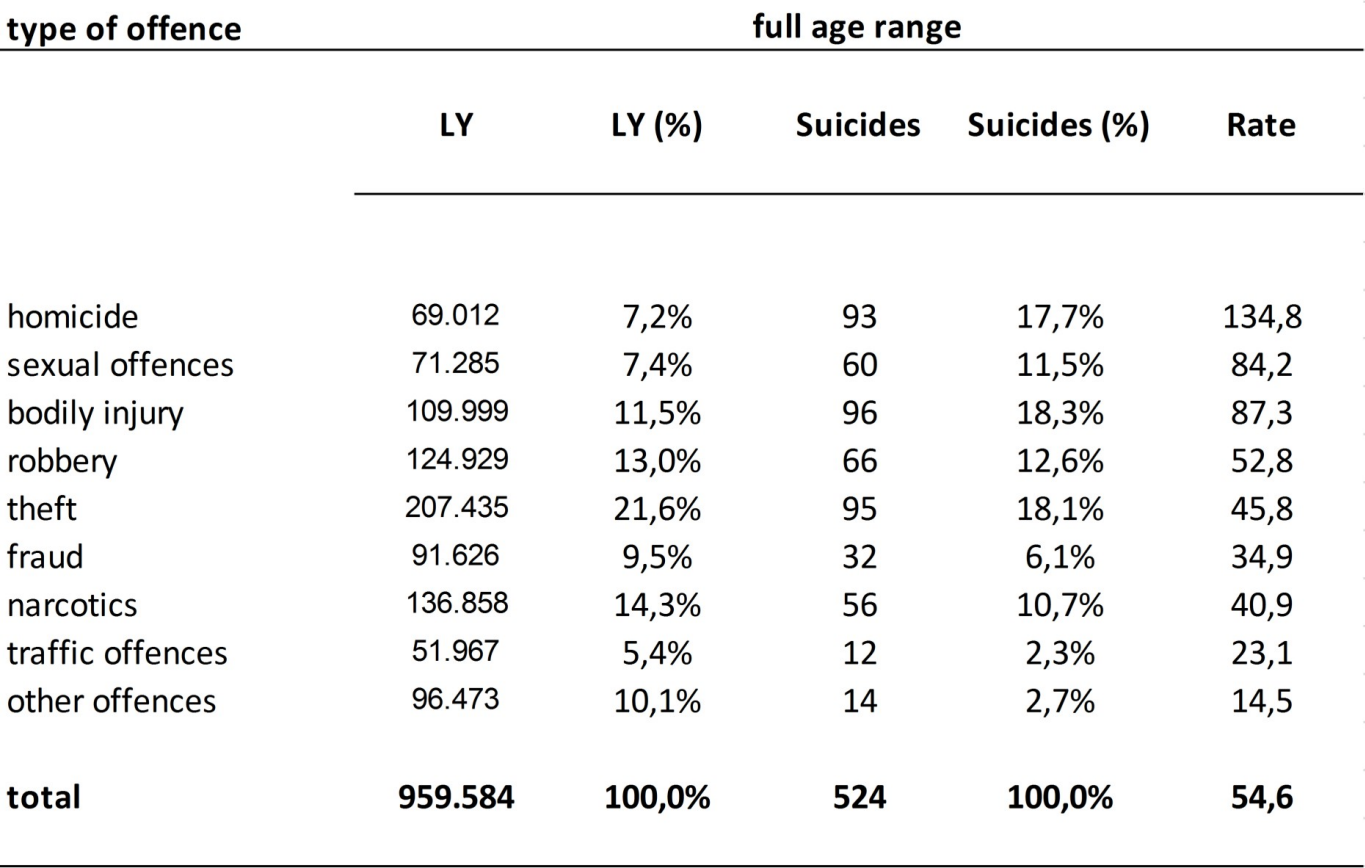
Number of individuals, suicides and suicide rates among offence related sub-populations in criminal detention (full age range). Rate = suicide rate = number of suicides per 100.000 LY.

**Fig 2:**
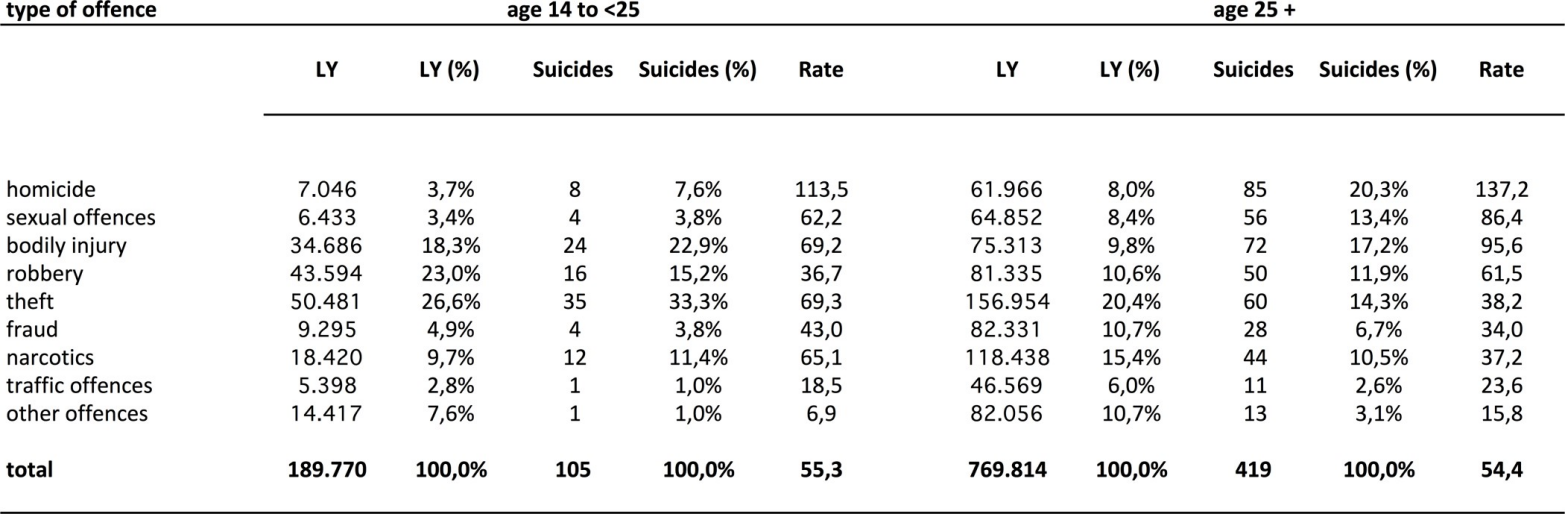
Number of individuals, suicides and suicide rates among offence related sub-populations in criminal detention given for adolescent and adults. Rate = suicide rate = number of suicides per 100.000 LY.

During the study period of 17 years, 959.584 LY were spent by male and 52.084 LY were spent by female prisoners in German criminal detention. From among those, 524 male and 14 female prisoners died by suicide. In the group of male prisoners, 189.770 LY were spent by adolescent prisoners and 769.814 LY by adult prisoners. 105 suicides were conducted in the adolescent group and 419 in the adult group. The age structure of the adolescent group was composed as follows: 5,7% minors, 27,9% 18–21 year olds, 66,4% 21–25 year olds. Within adolescents, suicide risk was higher for younger age groups. For detailed information on the age structure of prisoners and suicides in the prison population see Tables A, B, C in S1 Appendix. Figs 1 and 2 show frequencies for each offence related sub-population for the total male sample and for both age groups.

### SR in offence related sub-populations

The suicide risk of most offence groups differed significantly from the suicide risk of the overall prison population. Statistical characteristics for each offence group were given as follows. Homicide: X^2^[1; N = 1.029.213] = 68,86; *p* < ,001; sexual offences: X^2^[1; N = 1.031.453] = 10,23; *p* = ,001; bodily injury: X^2^[1; N = 1.070.203] = 18,15; *p* < ,001; robbery X^2^[1; N = 1.085.103] = 0,06; *p* = ,800; theft X^2^[1; N = 1.167.638] = 2,49; *p* = ,114; fraud: X^2^[1; N = 1.051.766] = 6,12; *p* = ,013; narcotics: X^2^[1; N = 1.097.022] = 4,24; *p* < ,039; traffic offences: X^2^[1; N = 1.012.087] = 9,24; *p* < ,002 and other offences X^2^[1; N = 1.056.595] = 27,66; *p* < ,001.

**Fig 3:**
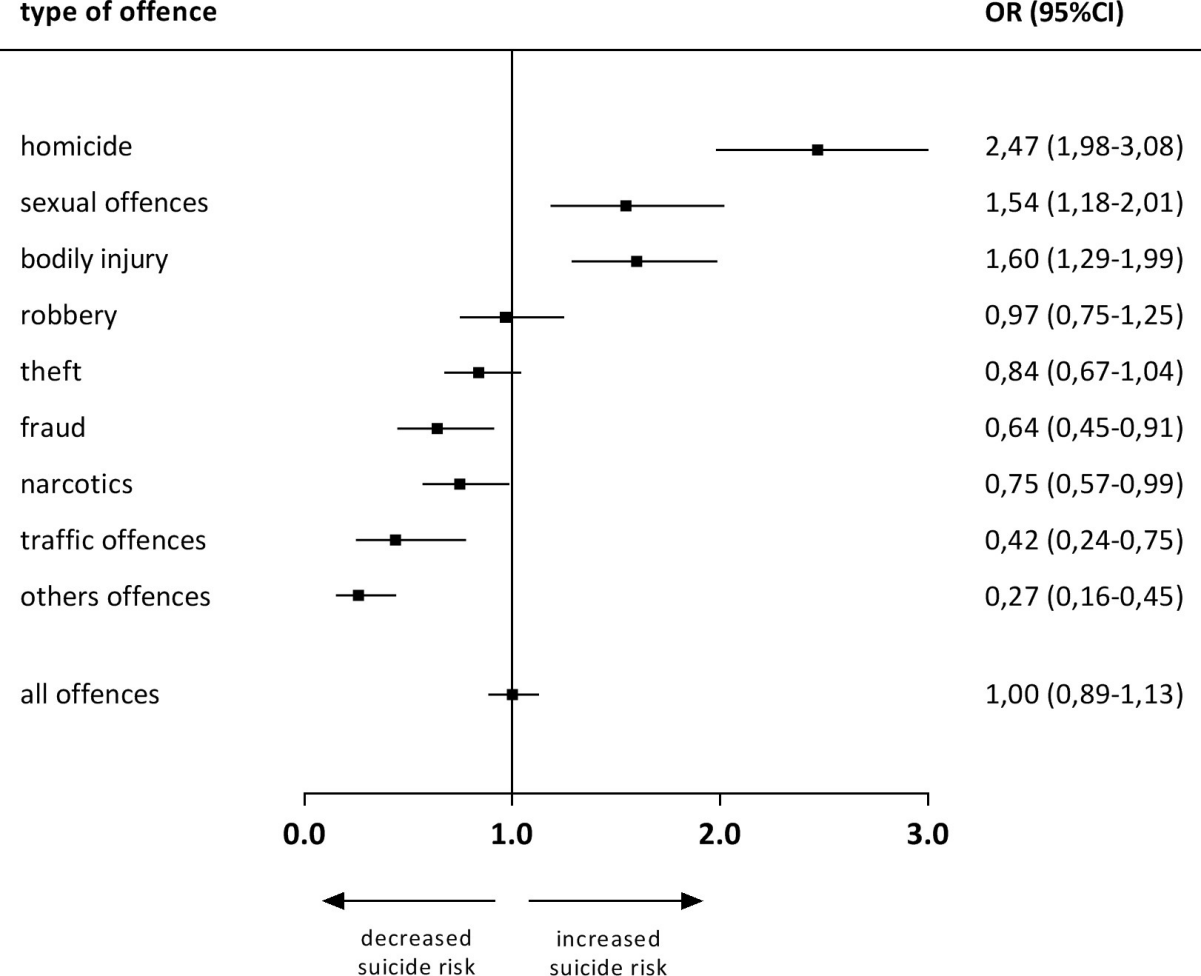
Suicide Risk (SR) in offence related sub-populations of prisoners compared to the average SR in the total prison population. Odd's ratios (OR) > 1 indicate a higher SR in the specific offence group compared to the total prison population. OR < 1 indicate a smaller SR in the specific offence group compared to the total prison population. Results are significant (p<0,05), if confidence intervals (CI) do not cross the line at which OR = 1,0.

**Fig 4:**
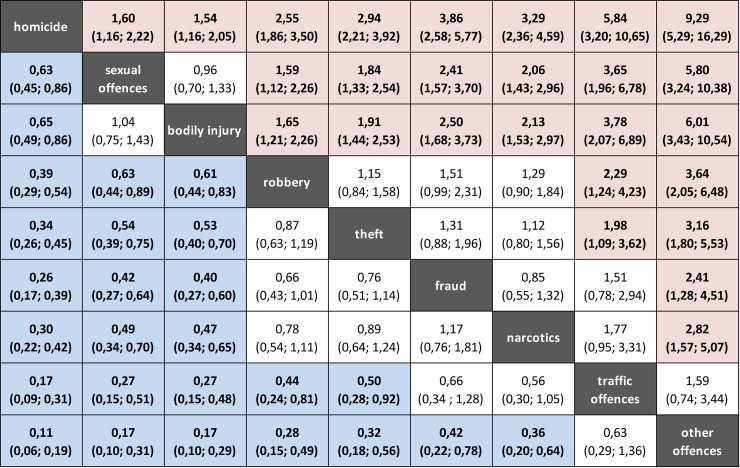
Head-to-head comparison of suicide risks (SR) of offence-groups. Entries in each cell represent the suicide risk ratio of the row-defining offence-groups compared to the column-defining offence-groups, given as Odd's Ratios and 95% confidence intervals. For example the suicide risk of prisoners convicted for homicide is increased by factor 1,60 compared with prisoners convicted for sexual offences; vice versa, prisoners convicted for sexual offences show a suicide risk diminished by factor 0.63 compared with prisoners convicted for homicide. Cells representing significantly increased (decreased) suicide risk were marked with red (blue) colour.

Fig 3 shows ORs and 95%CIs for each offence related sub-population in comparison to the average SR in the total prison population. Fig 4 presents direct comparisons of offence related SR. It shows that e.g. prisoners convicted for homicide have a 1,6 times higher risk to commit suicide while incarcerated than prisoners convicted for sexual offences.

### Comparison of adolescent vs. adult detainees

**Fig 5:**
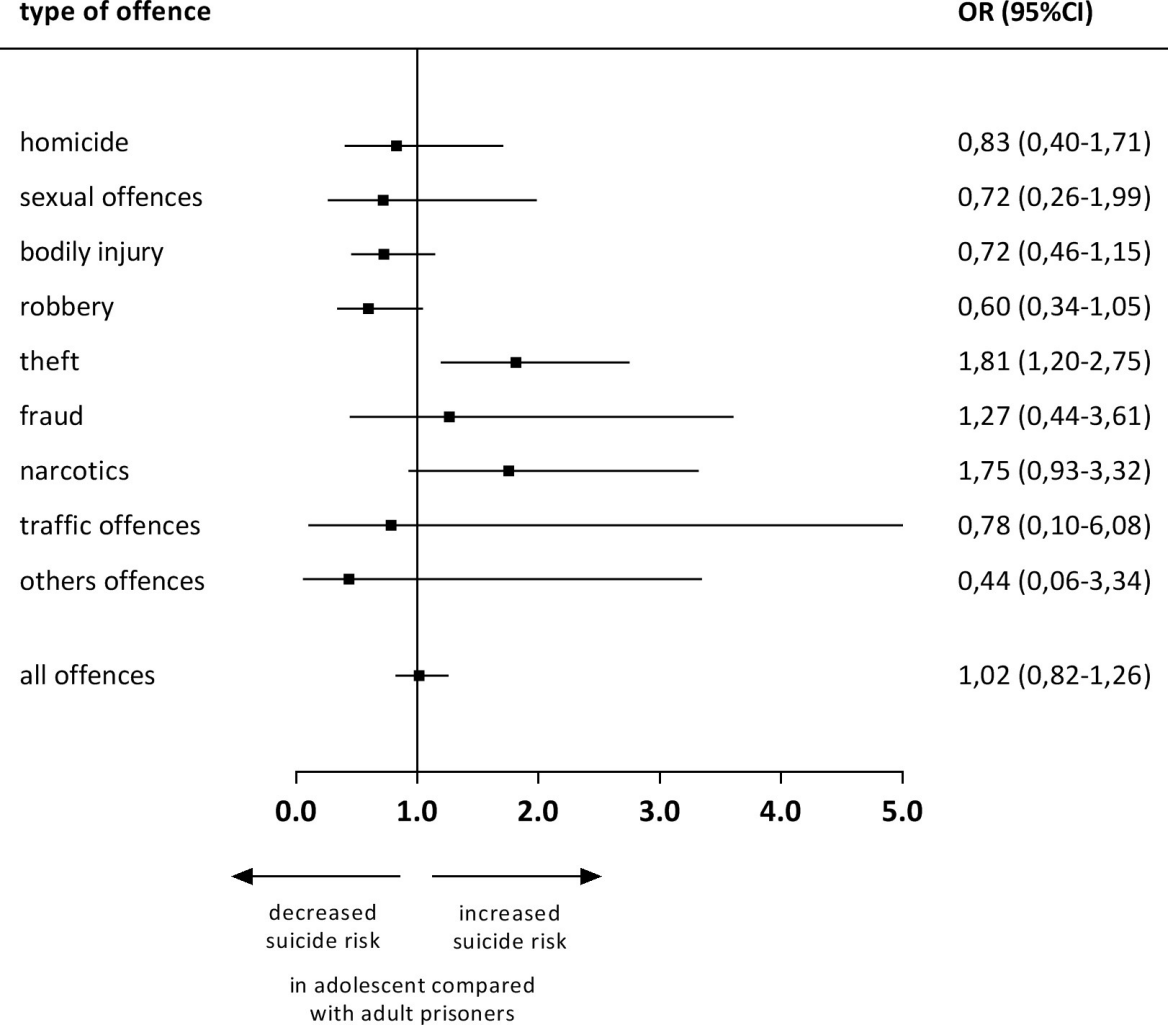
Offence related suicide risk (SR)—comparison of adolescent and adult age groups. Odd's ratios (OR) > 1 indicate an increase of SR of the adolescent offence related sub-population compared to the adult offence related sub-population. OR < 1 indicate a decrease of SR of the adolescent offence related sub-population compared to the adult offence related sub-population. Results are significant (p<0,05), if their confidence intervals (CI) do not cross the line OR = 1,0.

Results of the Tarone test for homogeneity indicated that the association between SR and age group was heterogeneous between offence groups (X^2^[8; N = 959.584] = 17,97; *p* = ,021). Post hoc tests showed significant differences for theft (X^2^[8; N = 207.435] = 8,07; *p* = ,004), and trending results (p<0.10) for robbery (X^2^[8; N = 124.929] = 3,29; *p* = ,069) and narcotics (X^2^[8; N = 136.858] = 3,06; *p* = ,081). Fig 5 shows OR and 95%CI for adolescents in comparison to adults for each offence group.

## Discussion

Suicide prevention in prisons is a multilevel approach, including key components like training and awareness programs of the prison staff, intake screening and on-going observation of suicide intent, improvement of architecture, and management following screening i.e. mental health treatment [2]. The aim of this study was to provide some evidence for risk assessment procedures in prisons. Since a large amount of suicides take place during the first weeks after intake, guidelines emphasize the need for risk assessment immediately after imprisonment [2].

There are two main findings resulting from this study: 1) SR differed between offence subgroups with highest risks for offenders convicted for offences against life, against bodily integrity or against sexual self-determination. 2) The offence-related SR for theft differed significantly between age groups with higher SR in adolescents. Trending, the SR for robbery and drug-related offences differed between adolescents and adult detainees, indicating higher SR for adolescents detained for drug-related offences and lower SR for adolescents detained for robbery.

Our results support hypothesis 1 (offence-related sub-populations show different SR with highest risks for homicide and offences against bodily integrity). This finding is consistent with previous studies reporting SR in British, French and Austrian prison populations [16,17,30]. Our study makes a significant contribution to the evidence base by reporting data from a large representative sample.

Potential contributing factors to higher SR in the mentioned offence groups are diminished levels of impulse control [31], experience and knowledge of bodily injury, long average terms of imprisonment, a high level of social disapproval of the offences, feelings of guilt and shame as most of physical offences are committed towards a family member. The latter factors may contribute to a loss of belongingness, perceived burdensomeness and hopelessness in violent offenders, all of which are described as predisposing conditions for suicide [32,33].

In order to carry out an age comparison of sufficiently large groups, the age-bandings 14–18 years, 18–21 years and 21–25 years were merged. As a result, this adolescent age group was a mixture of underage and young adult prisoners, with the age range of 21–25 year-olds accounting for about two-thirds of this population. In the age range 14–21 suicides were overrepresented in comparison to the total prison population (for details see Table A in S1 Appendix). This finding confirms the current WHO guidelines for suicide prevention in prisons, which define young prisoners as an important risk group [2]. Adolescents placed in adult prisons are most vulnerable [21]. In future meta-analyses, the specific risks of underage prisoners could be examined more closely.

With regard to hypothesis 2, significant differences in SR for adolescents and adults were identified for theft and trending differences for robbery and drug-related offences. The lack of further significant differences was due to particularly wide offence-related CIs for the SR of adolescent detainees, ensuing from the lower overall number of adolescent detainees within the respective offence groups.

Reasons for differences in offence-related SR between age groups remain speculative. Age-related challenges in detention [21,25,34] may contribute to this finding as well as differences between juvenile and adult criminal law. In Germany, juvenile criminal law applies for young offenders between 14 and 18 years. It is possible that courts convict offenders between 18 and 21 years of age according to juvenile criminal law subject to their emotional, mental, and intellectual maturity [35]. In the case of long prison sentences, these prisoners are transferred to the adult prison. Detention is the ultima ratio for juvenile offenders and as a result, adolescent prisoners are likely to show a higher number of risk factors like early delinquency, serial offences, exceptional seriousness of offences and high impulsiveness. Also, there is evidence from meta-analytic studies, that differences exist between adolescent and adult age groups regarding the association of suicide communication and suicide [36]. As a result, conclusions drawn from studies with adult detainees cannot be transferred to juvenile detainees without critical inspection.

Possible reasons why adolescent prisoners convicted for theft or offences against the narcotics law showed significantly higher SR than adults may be that theft is an offence that may lead to one of the first imprisonments of a young detainee. Therefore, it may increase the crisis aspect as a situational risk factor through novelty of experiencing drastic social changes. Furthermore, acquisitive crime with substance dependency may play a stronger role and may have a higher prevalence among adolescent offenders convicted for theft than for adults. Personal substance consumption and dependency may also be higher in adolescents than in adults convicted for drug-related offences.

Since the data available for the present study did not contain detailed context specific, individual or situational information on the case level, reasoning for differences between offence-related SR remains speculative. Our approach was limited by a retrospective and descriptive methodology. In order to improve the predictive value of risk assessments in German prisons, relevant risk and protective factors should be assessed during intake procedures and subsequent near lethal self-harm incidents and suicides should be documented. The predictive value of suicide risk factors needs to be evaluated in a group of prisons or on a national basis, therefore risk assessment and documentation should be standardized in order to merge data. With this approach, an evidence-based multi-risk assessment could be implemented in future. Nonetheless, we hope to support the valuable work of prison staff with this study by strengthening the evidence base for risk assessment during intake procedures.

## Limitations and strengths

Some limitations apply to this study. First, due to data structure, results were not corrected for possibly confounding variables (e.g. length of sentence or prison conditions). Second, small case numbers of suicides among adolescents limited statistical power further, so that wide confidence intervals impede the interpretation of differences between offence-related SR in age groups, especially in offence groups with a small number of detainees.

However, a particular strength of this study is that its results are based on a national sample of the full male German prison population in the study period. With a total sample of 959.584 LY for prisoners and 524 reported suicides, this is the largest population analysed in an original publication addressing this research question.

## Conclusion

In this total national study, we found that SR is not stratified homogeneously across offence types. Significantly higher SR were found in prisoners detained for an offence resulting in extensive physical harm for another person. To our knowledge, this is the first study that analysed differences in offence-related SR between adolescent and adult prisoners. Based on the results of this study, we propose that age- and offence-related risk factors should be assessed consistently in multidimensional suicide risk screenings in prisons.

## Supporting information

S1 Appendix Detailed information on life years spent in prison (Table A in S1 Appendix), suicides (Table B and Table C in S1 Appendix) and suicide rates (Table D in S1 Appendix).
(XLSX)
